# Acute Basilar Artery Occlusion and Death Secondary to Bilateral Vertebral Artery Dissection

**DOI:** 10.7759/cureus.27927

**Published:** 2022-08-12

**Authors:** Austin M Graf, Ilya Sakharuk, Peter D Drevets, Adil M Abuzeid

**Affiliations:** 1 Department of Surgery, Augusta University Medical College of Georgia, Augusta, USA

**Keywords:** spinal decompression, anti-thrombotic therapy, peri-operative medicine, head and neck trauma, stroke, basilar artery thrombosis, vertebral artery dissection

## Abstract

Vertebral artery dissection as a cause of basilar artery thrombosis is an exceedingly rare event that is associated with significant morbidity and poor outcomes. We present an unusual case of bilateral vertebral artery dissection and spinal cord compression in a 21-year-old male involved in a diving accident. The patient received limited antithrombotic therapy in pursuit of surgical spinal decompression, ultimately contributing to thrombosis of the basilar artery in the post-operative period and death following anterior cervical discectomy and fusion. Our goal is to highlight the severity of vertebral artery injury and the critical importance of treatment in the prevention of associated sequelae.

## Introduction

Vertebral artery dissection (VAD) is most commonly associated with blunt traumatic injury to the head and neck. The Modified Denver Screening Criteria is often used to determine when computed tomographic (CT) angiography of the neck is indicated in instances of blunt cerebrovascular injury [1]. The incidence of VAD is unclear, with the estimated occurrence of approximately 1.1/100,000 individuals in the United States [2]. The symptoms associated with VAD are often non-specific, ranging from headache to stroke-like deficits. Treatment of VAD primarily involves anticoagulation or anti-platelet therapy to prevent thrombosis and arterial occlusion. We present a case of bilateral VAD in a 21-year-old male after a diving accident with subsequent thrombosis of the basilar artery following anterior cervical discectomy and fusion (ACDF) for concurrent spinal cord compression.

## Case presentation

**Figure 1:**
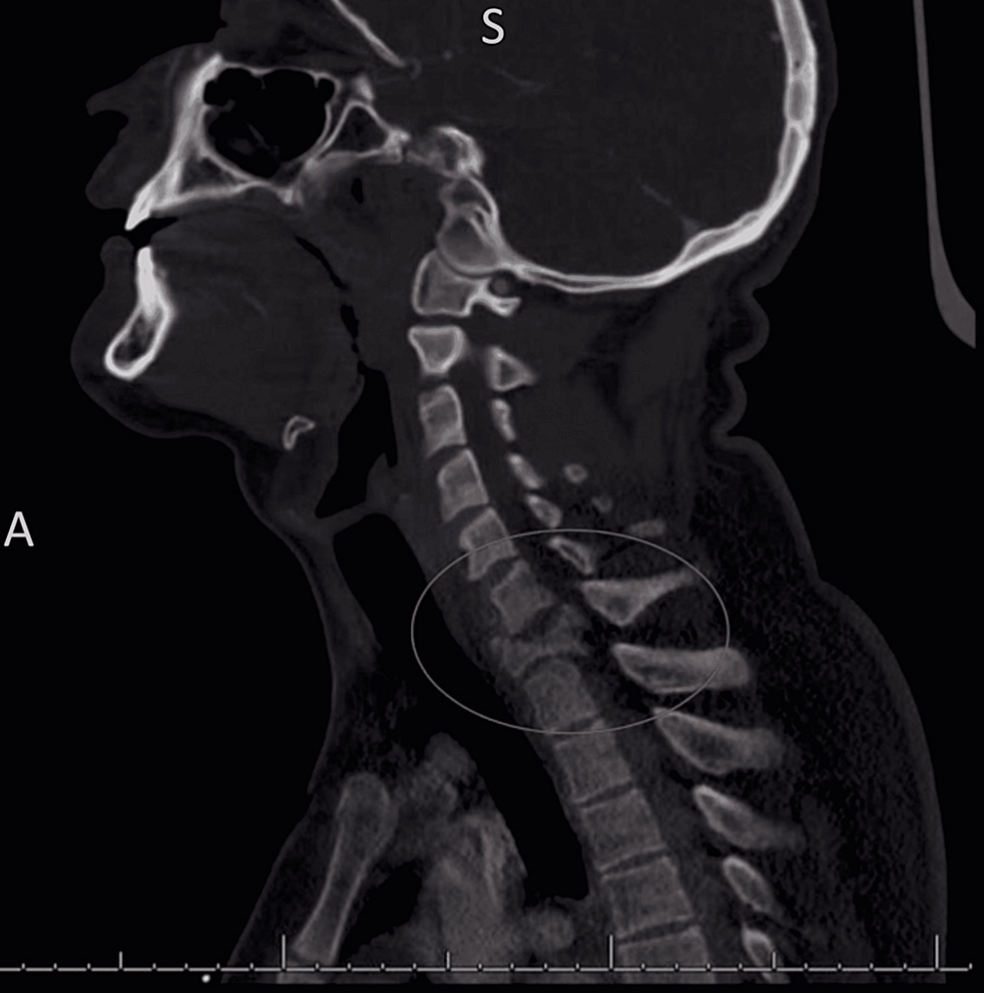
CT cervical spine without contrast showing unstable C7 burst fracture with retropulsion and severe cord compression. CT, Computed Tomography

A 21-year-old male with no significant past medical history was brought emergently to our Level 1 Trauma center after a diving accident in which he dove into shallow water, striking his head on the bottom of a lake. His blood pressure on arrival was 156/115 mmHg, pulse 60 beats·min^-1^, respiratory rate 17 breaths·min^-1^, and temperature 36.8°C. The patient’s primary survey was intact with a Glasgow Coma Scale (GCS) score of 15. On a secondary survey, the patient was found to have absent sensation and motor function below the level of T1. CT imaging was obtained of the patient’s head and cervical spine. CT head imaging revealed normal anatomy with no noted intracranial abnormalities, while CT neck imaging demonstrated an unstable burst fracture of the C7 vertebra with retropulsion and severe cord compression (Figure 1).

**Figure 2:**
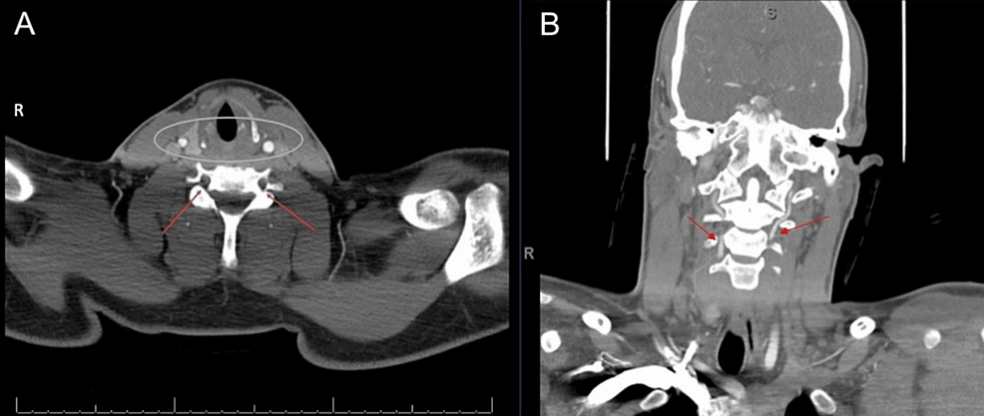
(A) CT angiography neck with contrast showing loss of contrast opacification of the bilateral vertebral arteries at the V1 (preforaminal)-V2 (foraminal) junction at the level of C5 consistent with bilateral vertebral artery dissection (arrows). No traumatic injury to the carotid arteries was seen (circle). (B) CT angiography neck with contrast showing reconstitution and normal contrast opacification and vessel caliber at approximately the C3 levels (arrows). CT, Computed Tomography

CT angiography was then obtained and showed evidence of bilateral VADs in the V2 (foraminal) segment (Figure 2A). There was segmental loss of opacification with reconstitution at the level of the C3 vertebra (Figure 2B).

The patient was admitted to the Trauma Intensive Care Unit and was started on acetylsalicylic acid (Aspirin, Bayer) 81 mg following Vascular Surgery evaluation. He remained in stable condition without any obvious change in his neurologic status. The Orthopedic Surgery service was consulted to evaluate the patient and elected to take the patient to the operating room for cervical arthrodesis in an attempt to decompress his cervical spinal cord with the aim of restoring his lower extremity function. Concern regarding the development of a post-operative spinal epidural hematoma resulted in holding all anticoagulation and any additional anti-platelet therapy.

**Figure 3:**
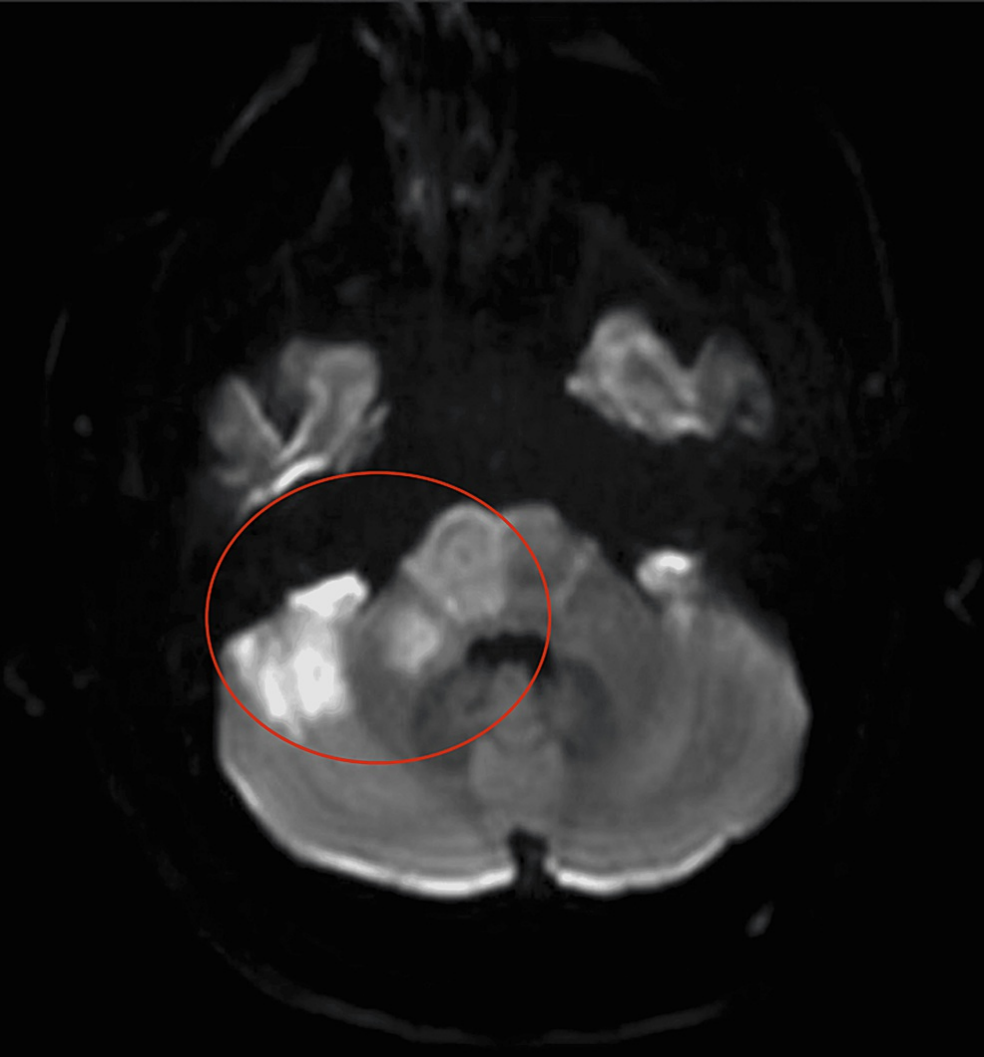
MRI brain without contrast showing ischemia/infarction within the right midbrain, right pons, right brachium pontis, and right cerebellar hemisphere (circle). MRI, Magnetic Resonance Imaging

Five hours after arrival, the patient underwent an uneventful C7 corpectomy with C6-T1 ACDF. In post-operative recovery, the patient was observed opening his eyes and moving his bilateral upper extremities. Three hours after the procedure, however, he was noted to be anisocoric. Emergent non-contrast and contrast CT of the head showed thrombosis of the basilar artery. Magnetic resonance imaging (MRI) confirmed basilar artery thrombosis and additional thrombosis of bilateral anterior inferior cerebellar arteries with ischemic changes and possible infarction of the pons, right midbrain, and bilateral cerebellar hemispheres (Figure 3). Goals of care were discussed with the family, who decided to proceed with comfort care only. The patient died soon afterward.

## Discussion

While VAD is generally seen following major trauma to the head and neck, it has also been reported to occur spontaneously secondary to intimal weakening from connective tissue disorders. In the United States, total incidence of VAD is estimated to occur in about 1.1/100,000 individuals [2]. The Modified Denver Screening Criteria (Table 1) is often used clinically to determine when CT angiography of the neck is indicated and highlights the importance of early detection through imaging in instances of blunt cerebrovascular injury [1]. Individuals with VADs typically report an array of symptoms including head pain, difficulty with speech, swallowing, coordination, balance, and other symptomatology associated with poor perfusion to the posterior hemisphere of the brain. It is estimated that stroke-like symptoms are seen in 77%-96% of patients, with 67%-85% of individuals experiencing ischemic stroke [2]. Advances in neurovascular imaging have allowed for an increased understanding of the complexity and variety of VADs. Dissections in the setting of trauma are associated with excessive force to the cervical spine, typically substantial enough to cause a fracture, as seen in our patient [3]. The mechanism of injury is related to the relatively limited mobility and fixation of the vertebral artery as it traverses the spinal column within the foramina of the vertebral bodies and enters the base of the skull [2]. In trauma, stress is focused on critical tether points along the artery instead of the entire vessel, leading to intimal dissection.

**Table 1:** Current Denver Screening Criteria for blunt cerebrovascular injury BCVI - Blunt Cerebrovascular Injury, TIA - Transient Ischemic Attack, CT - Computed Tomography, MRI - Magnetic Resonance Imaging, TBI - Traumatic Brain Injury Source: [1]

Signs and Symptoms
Potential arterial hemorrhage from the neck/nose/mouth
Cervical bruit in patients <50 years of age
Expanding cervical hematoma
Focal neurologic deficit (transient ischemic attack, hemiparesis, vertebrobasilar symptoms, Horner syndrome)
Neurologic deficit inconsistent with head CT findings
Stroke on CT or MRI
Risk Factors
High-energy injury plus:
Le Fort II or III displaced midface fracture
Mandible fracture
Complex skull fracture
Basilar skull fracture
Scalp degloving
Cervical spine fracture, subluxation, or ligamentous injury at any level
Severe traumatic brain injury with Glasgow coma scale <6
Near hanging with hypoxic-ischemic (anoxic) brain injury
Clothesline type injury or seat belt abrasion with significant swelling, pain, or altered mental status
Traumatic brain injury with thoracic injuries
Upper rib fractures
Thoracic vascular injuries
Blunt cardiac rupture

Once a diagnosis of VAD is established through imaging, expedited treatment is warranted to prevent thrombosis and ensuing infarction of the brain. Arterial dissection is proposed to cause ischemic events by two mechanisms - 1) thromboses forming at the location of the dissection and embolizing distally or 2) hemodynamic insufficiency related to the lack of blood flow distal to the injury. Most events of ischemia in this setting are thought to occur via the thromboembolic route and can be effectively prevented with prophylactic doses of anticoagulation or antiplatelet therapy, although this is still debated in the literature [1,3,4]. Treatment is generally started as soon as possible following diagnosis of VAD on imaging. In our case, the patient had a significant injury to the cervical spine and the decision to start anticoagulation was confounded by the need to emergently address his cervical cord compression via operative intervention.

One of the most severe complications of VAD is basilar artery thrombosis or acute basilar occlusion (ABO). Though an exceedingly rare event, ABO has been noted to occur following both traumatic and spontaneous cases of VAD. Additionally, limited literature exists investigating intraoperative ABO during ACDF. It is hypothesized that hyperextended positioning of the neck during surgery leads to a potentially harmfully low flow state in the vertebral arteries as the source of ABO [5]. Martin et al., in a similar case of ABO, states that the hyperextension of their patient’s neck during surgery led to the dissection of the vertebral arteries and stroke postoperatively [6]. Our patient defers from these case reports due to his vertebral arteries having already been dissected prior to surgery. Additionally, while in post-operative recovery the patient was observed with neurological function consistent with preoperative findings and it was not until three hours after the procedure that he was noted to have neurologic dysfunction. If the stroke was the result of the ACDF it would have likely presented intraoperatively or immediately postoperatively on withdrawal of anesthesia, as the low flow state to the vertebrobasilar arteries was ameliorated upon completion of surgery. While ACDF cannot be completely ruled out as a contributor to the ABO in our patient the delay of the event until significant postoperative time had passed makes VAD a much more likely precipitating event. Prognosis following basilar artery thrombosis is dismal, with patients typically experiencing significant neurological symptoms. Recovery following such an event is rare and typically occurs when the event is identified immediately, before the development of widespread cerebral ischemia.

Management of ABO has traditionally included the use of anticoagulant medications and thrombolytics. There are few large-scale studies comparing management strategies of ABO and most evidence is derived from case series and stroke intervention trials. Schonewille et al. retrospectively assessed the outcomes and treatment responses in over 600 patients who presented with confirmed basilar artery occlusion [7]. The study looked at the outcomes of patients receiving antithrombotic agents, primary intravenous thrombolysis, or intra-arterial therapy. The results were equivocal, showing no superiority for any therapeutic strategy with 68% of patients having poor outcomes, including death, despite treatment. The association of VAD as a preceding injury and the high likelihood of mortality following ABO stresses the importance of prevention through early prophylactic anti-thrombotic therapy [1,4].

With the advent of new endovascular therapies and techniques, endovascular extraction of a basilar thrombus has shown promise. There are several case reports in the literature showing remarkable recovery, with patients experiencing only minor sensory and cognitive deficits months after the insult [8]. There are currently no studies available comparing the efficacy of endovascular therapy to traditional treatment with medication, and so antiplatelet and anticoagulation medications remain the mainstay treatment of basilar artery thrombosis, with thrombolytic therapy offered on a case-by-case basis.

## Conclusions

The patient described in our report had a severe vascular injury that was complicated by significant trauma to the cervical spine. Bilateral VAD in the context of severe spinal injury has seldom been reported and no major trials exist to guide the management of these patients. Our case highlights the importance of increased consideration for primary anticoagulation or antiplatelet therapy in VAD, even in the event of multiple injuries, specifically spinal cord compression and loss of neurologic function, as the risk of thrombosis and associated sequelae, including death, remains high. High clinical suspicion should exist in cervical trauma as dissection of the vertebral arteries can occur due to their relative immobility. CT imaging is likely the most appropriate imaging modality due to ease of access and adequate visualization of the involved vessels. Once the diagnosis of VAD is made, anti-thrombotic therapy should be prioritized to prevent subsequent thrombosis as the risk of ABO increases in the setting of VAD. Although current literature has not reached a consensus regarding anticoagulation vs anti-platelet therapy, unfractionated heparin prophylaxis may be preferred over low molecular weight heparin or aspirin due to the availability of protamine reversal. In the event of ABO, interventions have proven to have limited efficacy with an overwhelming percentage resulting in mortality.

Our patient’s stable presentation and young age made determining a course of action difficult. Although he received aspirin after VAD was recognized, more extensive anticoagulation or antiplatelet therapy was held in anticipation of his cervical arthrodesis, almost certainly contributing to the outcome. Further studies should be conducted to determine the best treatment course in VAD and ABO in the setting of multiple traumatic injuries and post-operatively - cases in which anticoagulation and thrombolysis are made risky and are therefore rarely attempted. The development of basilar artery thrombosis remains a rare event with a poor prognosis in which treatment options are limited.
